# Operative Treatment of Acute Intestinal Obstruction

**Published:** 1885-10

**Authors:** 


### THE OPERATIVE TREATMENT OF ACUTE INTESTINAL OBSTRUCTION.

FREDERICK TREVES, F. R. C. S., Hunterian Professor at the Royal College of Surgeons of England, etc., has an elaborate paper on this important subject in the *N. Y. Medical Record* of August 15.

Intestinal obstruction is a vague expression, and may have different meanings to different minds—and may embrace a wide range of cases totally dissimilar as to causation, and alike only as to pathological results. He says the operative treatment of intestinal obstruction should be considered under three heads: 1, The treatment of acute obstruction; 2, The treatment of chronic obstruction, and 3, The treatment of chronic cases that have become acute; but for want of space and time, he limits this paper to discussion of the treatment of the acute form, "since it presents the most urgent claims to the surgeon's attention." After discussing the various forms of acute obstruction, and the various plans adopted for treatment; and after cautioning against "masking the symptoms" by opiates before a clear diagnosis has been made, the author says:

"It must be evident, however, that these meager data form no excuse for delay. Laparotomy is, in itself, not a serious undertaking. Its high mortality in the present class of cases depends, I venture to believe, upon the fact that the operation is usually undertaken too late. It is regarded as a last resource, whereas it should be the first resource, since it is the only resource. When once the diagnosis of strangulated hernia has been established, and taxis has failed, no surgeon, I imagine, is disposed to temporize. The condition of the bowel in these cases (acute obstruction) is identical with that found in strangulated rupture, and the therapeutic principles that apply to the one, should apply to the other. It seems to be tampering with life to waste time over the administration of metalic mercury and enemata of tobacco and the like. To thrust an aspirator into the abdomen, as some advise, is a stab in the dark, an empirical proceeding that leaves everything to chance. Massage, or abdominal taxis, has its advocates, but the procedure is, at the best, but a blind one. The manipulation of the abdomen may, by a rare combination of circumstances, reduce a snared loop, but it is as likely to aggravate its condition, and to produce a perforation in a segment of intestine that is approaching gangrene and that needs the tenderest handling. Even if the diagnosis be ill founded, the laparotomy merely resolves itself into an exploratory incision. Such incision adds but a fractional part to the sum total of the risk to life involved; it displays a course of action, and even if it be found to be of no avail, it is questionable whether in many cases it hastens the inevitable ending. The use of laparotomy in other than acute intestinal diseases for purely diagnostic purposes has been clearly established, and has been accredited a position, and undoubted value.''

After reviewing all the points for and against the operation, the author closes the discussion as follows :

''In favor of the operation it must be pointed out that the affection is very acute; that the general mortality of the disease is seventy per cent, and that eight per cent. of the patients die before the seventh day. I would venture to urge that in these acute cases, laparotomy should be performed at least within the first forty-eight hours, and if possible, within the first twenty four hours, provided, of course, that all other measures have failed.'' [That is contradictory; he has just advised not to waste time on other measures—and instances metalic mercury and tobacco enemata as useless—and also condems massage.—Ed.]

